# Anesthetic Gases Do Harm to the Environment, Is It Time for a Change?

**DOI:** 10.5152/TJAR.2022.7222

**Published:** 2022-02-01

**Authors:** Özge Köner

Climate change is a global health burden which is expected to cause approximately 250 000 additional deaths per year between 2030 and 2050.^1^ Inhaled anesthetic agents undergo minimal metabolism, therefore expelled to the atmosphere unchanged. Yearly emission of inhaled anesthetic agents in the USA was estimated to be equal to the annual emissions of one million automobiles in a year.^2^ Furthermore, the impact of inhaled anesthetics on global warming is on the rise since 1999, it rose from 0.003% to 0.6% in present day.^3^

An inhaled anesthetic agent’s contribution to global warming is referred to as its “carbon footprint”. This effect is measured by the global warming potential (GWP) of the gas and the equivalent CO_2_ mass is taken as a reference. Carbon dioxide equivalency (CDE) of a gas is measured by multiplying the mass emitted to the atmosphere (kg) and the known GWP of the agent.

The atmospheric lifetime of N_2_O is the longest (114 years) among the other inhaled anesthetic agents, and is followed by Desflurane, which has an 8.9 to 21 years lifetime. Isoflurane and Sevoflurane have relatively shorter lifetimes in the atmosphere being 2.6-5.9 and 1.1-5.2 years, respectively. However lifetime calculating methods are also changing (improving) and different data are presented in the literature. Intergovernmental Panel on Climate Change (IPCC) and World Meteorological Organization (WMO) are responsible for periodic evaluations and reporting of greenhouse gas emissions. The assessment is based on GWP as a reference. However latest evaluations about Sevoflurane have not been reflected on these organizations’ reports yet. It has been reported that GWP100 of Sevoflurane was updated as 127, which is 31-41% lower than reported before.^4^ Nitrous oxide (along with Isoflurane) is an ozone-depleting anesthetic gas, which has a significant GWP as the third most responsible greenhouse gas after CO_2_ and methane.^5^

Considering the negative impact of anesthetic waste gases on the atmosphere, taking some preventive measures is of utmost importance. For that purpose, some hospitals establish multidisciplinary operating room teams, which aim to reduce anesthetic gas waste. Another preventive way proposed is to use a two-pronged approach, which necessitates setting cumulative emissions limits for gases with longer half-life, and a maximum future emissions rate for the ones with shorter half-life. Low flow anesthesia has also been shown to reduce volatile anesthetic gas waste significantly.^6^ Greenhouse effects of Sevoflurane (2%), Desflurane (6%), Isoflurane (1.2%) and N_2_O (66%) are reduced by 75% when fresh gas flow is decreased from 2 L/min to 0.5 L/min (seven hours of use to reflect one day long general anesthesia with inhalational agents). Avoiding N_2_O as a carrier gas and using oxygen-air mixture instead may also reduce negative environmental effects of inhaled anesthetics. Automated control of the end-tidal inhaled anesthetics reduced the greenhouse gas effects by 44% compared with manual control. A new photochemical exhaust system helps destruct waste anesthetic gases of Desflurane and Sevoflurane efficiently at flow rates ranging from 0.25 to 2.0 L/min. However, removal of N_2_O is not as efficient, therefore requires further optimization. Strategies similar to those mentioned above may reduce the negative impact of inhaled anesthetic agents on the climate change. Those strategies need not be costly they can even be cost effective. 

It is us anesthesiologists’ duty along with other responsible parties to reduce greenhouse effects caused by anesthesia practice. Preferring low fresh gas flow rates (≤ 1 L/min), EEG guided inhaled anesthetic use, total intravenous anesthesia, regional anesthesia techniques are the measures to reduce footprint of anesthesia. Further studies on the real effects of inhaled anesthetics on the atmosphere using new technologies are warranted.
